# Task-Related Changes in Functional Properties of the Human Brain Network Underlying Attentional Control

**DOI:** 10.1371/journal.pone.0079023

**Published:** 2013-11-04

**Authors:** Tetsuo Kida, Ryusuke Kakigi

**Affiliations:** 1 Waseda Institute for Advanced Study, Waseda University, Tokyo, Japan; 2 Department of Integrative Physiology, National Institute for Physiological Sciences, Okazaki, Japan; Hospital General Dr. Manuel Gea González, Mexico

## Abstract

Previous studies have demonstrated task-related changes in brain activation and inter-regional connectivity but the temporal dynamics of functional properties of the brain during task execution is still unclear. In the present study, we investigated task-related changes in functional properties of the human brain network by applying graph-theoretical analysis to magnetoencephalography (MEG). Subjects performed a cue-target attention task in which a visual cue informed them of the direction of focus for incoming auditory or tactile target stimuli, but not the sensory modality. We analyzed the MEG signal in the cue-target interval to examine network properties during attentional control. Cluster-based non-parametric permutation tests with the Monte-Carlo method showed that in the cue-target interval, beta activity was desynchronized in the sensori-motor region including premotor and posterior parietal regions in the hemisphere contralateral to the attended side. Graph-theoretical analysis revealed that, in beta frequency, global hubs were found around the sensori-motor and prefrontal regions, and functional segregation over the entire network was decreased during attentional control compared to the baseline. Thus, network measures revealed task-related temporal changes in functional properties of the human brain network, leading to the understanding of how the brain dynamically responds to task execution as a network.

## Introduction

A large body of studies in neuroscience have investigated task-related changes in activation of different brain regions to infer functional specialization. Recent studies have extensively examined task-related changes in connectivity among the different brain regions [1]–[7]. Very recently, studies started to demonstrate how the brain works as a functional network or a set of sub-networks using functional magnetic resonance imaging (fMRI) [8]–[13] and magnetoencephalogaphy (MEG) [14]–[20]. Network measures most commonly used in these studies are derived from graph-theoretical analysis [21]–[23]. The clustering coefficient is a network measure of functional segregation primarily quantifying the presence of interconnected groups of brain regions, whereas betweenness centrality is a measure of centrality (global hub), which is considered to act as an important control of information flow [22]. Most of these studies using network measures examined functional properties of the brain network in a resting state, i.e., the default-mode network [14]–[15], [19], yet task-related temporal changes in functional properties of the human brain network remain unclear.

Here we used MEG to examine task-related temporal changes in functional properties of the human brain network. To this end, we used graph-theoretical analysis to compute network measures from the MEG signal recorded in a multisensory cue-target attention task. Our primary interest was to examine whether graph-theoretical analysis of MEG can detect task-related changes in network properties. Previous studies using fMRI have found the involvement of prefrontal and posterior parietal areas in the attentional control system [24]. Studies using MEG have shown that orienting attention to an upcoming sensory event modulates beta and alpha oscillations [25]–[28]. Beta desynchronization is also found in parietal areas following Knowledge of Results stimulation in MEG and electroencephalography (EEG) [29]. It also seems that there is a dichotomy between sensory and motor attention. Previous studies using fMRI and transcranial magnetic stimulation (TMS) have demonstrated the involvement of premotor and parietal regions in motor attention [30]–[31]. Beta desynchronization in EEG and MEG was also found in the contralateral sensori-motor region during movement preparation [32]. We thus hypothesized that beta oscillation is observed in the cue-target interval which requires the control of attention to stimulus and action. We also hypothesized for the network measure that global hubs are found in the prefrontal and sensori-motor regions involved in the control of attention to stimulus and action. Modulation of oscillation and the presence of global hubs (betweenness centrality) in the cue-target interval of this task may also provide information about the supramodal attentional control system [33]–[34] because we used a multisensory attention task to induce task-related changes in network measures.

## Materials and Methods

### Subjects

Recordings were obtained from nine healthy right-handed subjects (one woman and eight men), aged 24 to 52 years old. All subjects gave written informed consent prior to the study, which was first approved by the Ethics Committee of the National Institute for Physiological Sciences.

### Stimulation

**Figure 1:**
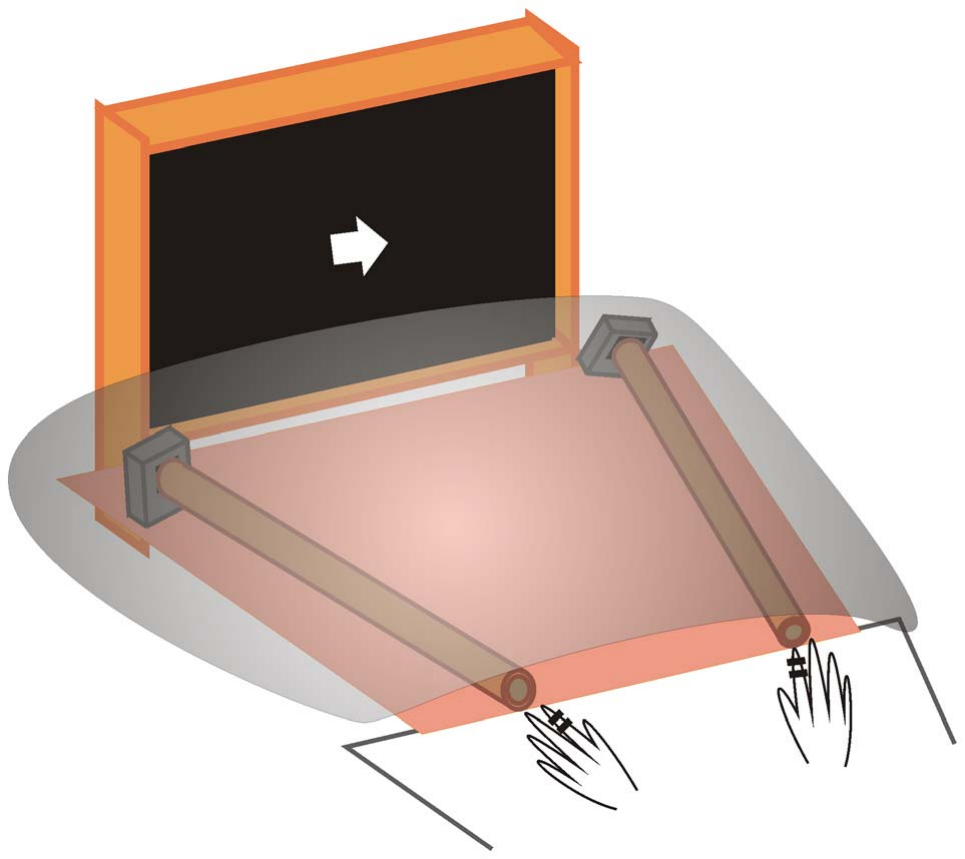
Stimuli used in this study.

A visual cue stimulus was followed 1.0–1.5 s later by an auditory or tactile stimulus. The interval between successive cue stimuli varied randomly between 4 and 5 s. Both auditory and tactile stimuli were used to examine the multisensory nature of task-related changes in the network properties. The cue stimulus was a right- or left arrow, which was presented in random order and with an equal probability on a screen through a digital light processing projector in front of the subjects at a distance of 2 m. The auditory or tactile stimulus had equal probability of presentation on the cue side or the opposite side, irrespective of the direction of the cue stimulus. The tactile stimulus was a single-pulse or double-pulse electrotactile stimulus presented in random order to the second digit of the left or right hand through ring electrodes (the cathode was attached to the proximal part of the finger, and the anode to the distal part). The stimulus lasted 0.2 ms and its intensity was 2.5 times the sensory threshold. In the case of the double-pulse stimulus, two electrotactile pulses separated by an interval of 150 ms were applied. The auditory stimulus was a single or double click presented in random order to the left or right side from card-type speakers (WM-R57A; Panasonic, Japan) through cylinders with a diameter of about 10 cm, made of reinforced cardboard (Figure 1). One end of each cylinder was placed 50 cm from the subject's nose, and the opposite end was fixed to the speakers. The surface of the cylinders was covered with soundproof material. The speaker was made of a piezo ceramic diaphragm to reproduce auditory tones without the use of a coil or magnet. The intensity was about 70dB/SPL when measured at the center of the MEG sensor array. This apparatus enabled perfect discrimination of the direction of clicks (i.e., left or right), and induced few magnetic artifacts, although that the auditory stimulus was presented close to the sensor array.

The subjects were instructed to placed the second digit of each hand near the auditory stimuli (i.e., near one end of the two cylinders) before the measurement started. They were instructed to attend covertly to the side of the visual cue, and to press a button when a double-pulse or double click was presented there. The to-be-attended stimulus and action sides were compatible in space, i.e., the responding hand was the hand on the attended side, to exclude the effect of stimulus-response incompatibility. Each subject received 960 trials (about 400 single-pulse electrotactile, 400 single-pulse auditory, 80 double-pulse electrotactile, and 80 double-pulse auditory trials, presented equally on left and right sides), divided into 12 blocks. Each block lasted about 4 minutes, and an experiment lasted about 50–60 minutes.

### Recordings

The MEG signal was recorded with a helmet-shaped detector array (Vectorview; ELEKTA Neuromag Oy, Helsinki, Finland), which in each of 102 locations has 3 sensors (306 sensors in all), two orthogonal planar gradiometers and one magnetometer coupled to a multi-SQUID (superconducting quantum interference device). Signals were filtered through a bandpass filter of 0.03–200 Hz and digitized at a sampling rate of 1,004 Hz. The subjects' head location relative to the MEG sensors was measured before the measurement using head position indicator coils. Eye movements and blinks were monitored with a near infrared camera (ISCAN; ISCAN Inc., Massachusetts, U.S.A.).

### Analysis

Data analyses were performed in Matlab (MathWorks) using custom scripts and open source toolboxes: Fieldtrip (http://fieldtrip.fcdonders.nl/) and Brain Connectivity Toolbox (BCT) (https://sites.google.com/a/brain-connectivity-toolbox.net/bct/). We analyzed the MEG signals recorded from 102 pairs of 2 orthogonal planar-type gradiometers. Line noise removal was performed (60, 120, 180 Hz components). The data were segmented from −2.5 sec before to 1.5 sec after the target stimulus. Trials with extremely high variance containing jumps and muscle artifacts were removed. Spectral analyses were performed using multitaper spectral estimates based on slepian sequences. We computed spectral estimates across logarithmically scaled frequencies *f* from 4 to 180 Hz and across 61 points in time *t* from −2.0 to 1.0 sec (0.05 sec step). The interval of logarithmically scaled frequencies was adjusted for equal spacing in time and frequency domains so that the number of frequency bins was 61. We adjusted the spectral smoothing to a 0.6 octave step, which means that spectral smoothing increases as the frequency of interest increases. The data for two orthogonal planar sensors were summed, resulting in a time-frequency representation at each of the 102 locations. Statistical analysis of the data segment was performed before target stimuli by means of cluster-based non-parametric permutation tests with the Monte-Carlo method (attend-left versus right conditions, 500 randomizations, significance level = 0.05).

In time-frequency analysis, beta desynchronization was observed during a cue-target interval. We thus focused on the beta frequency (center frequency, 16 Hz±4) to compute the phase locking value (PLV) [35] for all pairs of planar sensors as a measure of connectivity. The connectivity matrix obtained was subjected to graph theoretical analysis to examine task-related changes in network properties. We computed the betweenness centrality (measure of centrality) and averaged clustering coefficients over the entire network (measure of functional segregation) [21]–[22]. Betweenness centrality, *b_i_* of node *i*, is defined by the following equation,
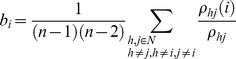
where *N* is the set of all nodes in the network, *n* is the number of nodes, *ρ_hj_* is the number of shortest paths between *h* and *j*, and *ρ_hj_* (*i*) is the number of shortest paths between *h* and *j* that through *i*. The averaged clustering coefficient *C* of the network is defined by the following equation,
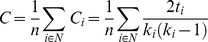
where *C_i_* is the clustering coefficient of node *i* (*C_i_* = 0 for *k_i_*<2), *k_i_* is the degree of node *i*, and *t_i_* is the number of triangles around node *i*,
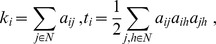
where *a_ij_* is the connection status between *i* and *j*: *a_ij_* = 1 when a link (*i*, *j*) exists (when *i* and *j* are neighbors); *a_ij_* = 0 otherwise (*a_ij_* = 0 for all *i*). (*i*, *j*) is a link between nodes *i* and *j*. These measures were calculated in two segments of 2000 ms before target stimuli in each of the attend-left and right conditions to compare pre-cue (baseline) and cue-target (attentional control) intervals. For the averaged clustering coefficient, two-way analysis of variance was performed with attention (baseline vs. cue-target intervals) and attended hemifield (right and left hemifield) as factors. Mauchly's sphericity test was also performed. We did not compute the characteristic path length, because this measure is not easy to interpret when computed from functional data [22].

## Results

### Spectral analysis

**Figure 2:**
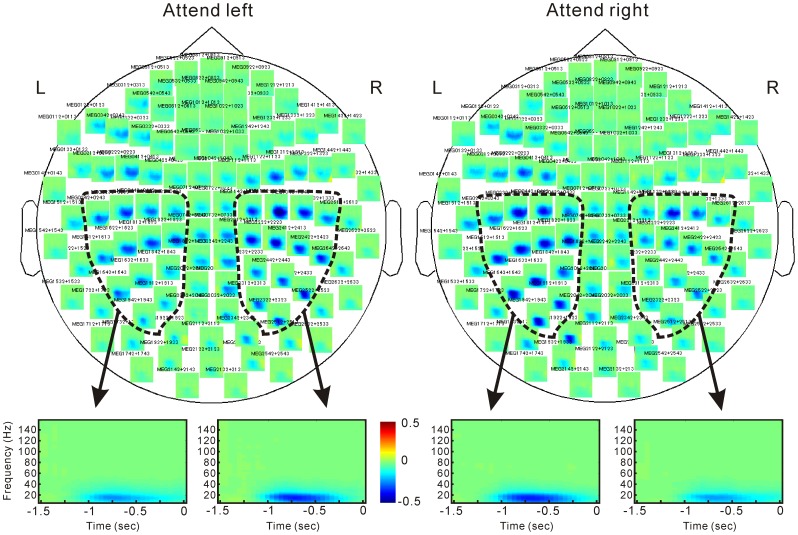
Time-frequency analysis. Zero on the horizontal axis indicates the onset of target stimuli. Data are expressed in color-coded images as a change relative to the baseline interval. Power increases and decreases are shown in red and blue, respectively.

**Figure 3:**
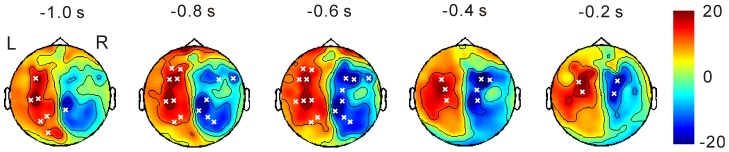
Cluster-based non-parametric permutation tests for attend-left versus right conditions on beta oscillation. Positive and negative clusters are shown in red and blue, respectively. Significant clusters are shown with a marker ‘x’. Positive clusters mean a power increase in attend-left versus right conditions, and negative clusters, power increases in the opposite contrast.

Time-frequency analysis showed that beta desynchronization continued from 1000 msec before and up to the target stimulus (Figure 2). Cluster-based permutation tests under attend left versus right conditions in the beta frequency found significant positive and negative clusters in the sensori-motor regions, including premotor and posterior parietal regions (Figure 3). This indicates a power decrease in the right hemisphere under the attend-left versus right conditions and that in the left hemisphere in the opposite direction. Thus, beta oscillation was more desynchronized in the hemisphere contralateral to the attended side.

### Network measures

**Figure 4:**
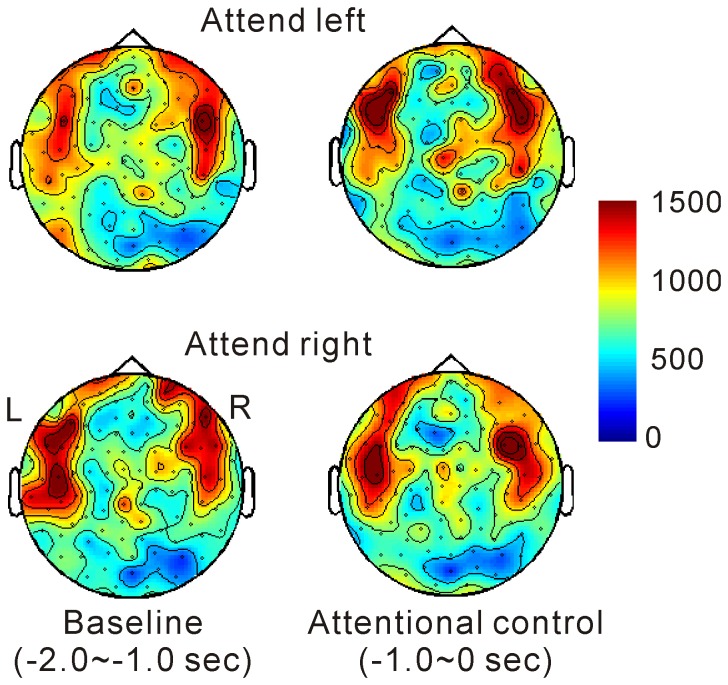
Betweenness centrality (global hub). Higher centrality was observed in the sensori-motor and prefrontal regions throughout the periods tested.

**Figure 5:**
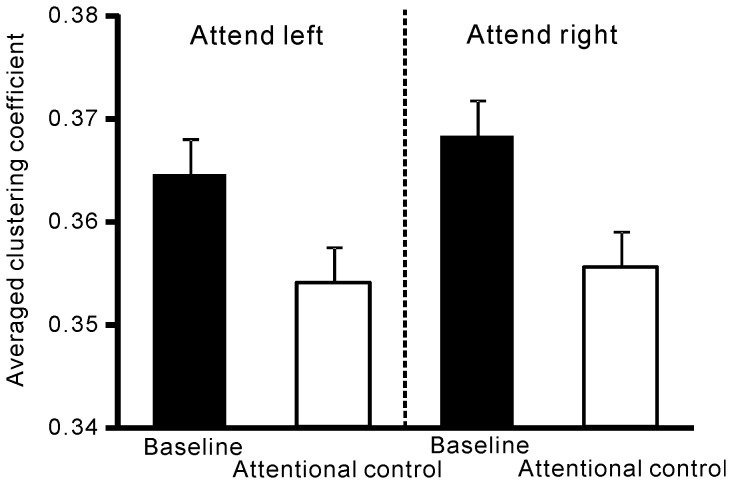
The averaged clustering coefficient over the entire network (functional segregation). The averaged clustering coefficient decreased during attentional control versus the baseline interval.

Global hubs reflected by betweenness centrality were found in the bilateral sensori-motor and prefrontal regions and remained stable over time (Figure 4). The averaged clustering coefficient over the entire network was significantly reduced in the cue-target interval compared to the baseline (Figure 5), as evidenced by a main effect of attention in two-way ANOVA (F (1, 8) = 10.6, *P*<0.05). There was no interaction between the two factors.

## Discussion

We found task-related temporal changes in network measures in a cue-target attention task using MEG. Functional segregation was decreased during attentional control versus the baseline interval, whereas global hubs found in the prefrontal and sensori-motor regions remained relatively stable over time. Previous fMRI studies have reported functional properties of the resting state brain network [8]–[9]. MEG studies have also demonstrated a variety of characteristics of the brain network in healthy individuals and patients [15], [17], [19], [36]. A recent study demonstrated the properties of the resting state brain network on MEG recordings, with global hubs being in the medial temporal lobe [14]. In contrast, the present study investigated task-related temporal changes in functional properties of the brain network, especially in the averaged clustering coefficient, and stability of global hubs in the cue-target interval. Thus, the present study can be the most fundamental first step to examine task-related rapid changes in functional network structures associated with a variety of brain functions.

Three possible systems for the organization of spatial attention have been proposed: 1) a completely supramodal attentional system which acts on any sense, 2) a completely unimodal system where attention acts individually on each sense, and 3) a separable but linked system where modality-specific attentional systems interplay across different senses [33]–[34], [37]–[39]. Global hubs found in the cue-target interval might be associated with the attentional control system. The source of the attentional control signal is considered to originate from frontal and parietal areas [24], [40]–[42], consistent with the present result. In addition, we have previously provided evidence for a separable but linked system by investigating attentional modulation of evoked responses in a visual-tactile cross-modal sustained attention task [39], [43]. We thus propose a hybrid system including a separable-but-linked system for stimulus processing and an attentional control system. A similar model was proposed in a previous event-related potential study [33], but our results extend this idea to network properties.

Modulation of beta oscillation was also found in the cue-target interval, which requires the control of attention to both stimulus and action. In the present study, the to-be-attended stimulus and action sides were compatible, excluding the effect of stimulus-response incompatibility. Thus, the beta desynchronization observed here may include the effects of attention to both stimulus and action. Studies of attention have found slow EEG deflections [44]–[45] and a power decrease of beta oscillations on MEG recordings [1], [25]–[26] during attentional control. Furthermore, recent studies have shown that spatial attention to tactile stimuli robustly modulates 10 Hz oscillations in the somatosensory cortex [27]–[28]. These are consistent with the present oscillation measurements. During movement preparation, beta desynchronization is observed in the contralateral sensori-motor region, which may be associated with non-specific pre-activation, priming or presetting of neurons in motor-related areas [32]. Also, TMS studies have shown the involvement of premotor and parietal cortices in motor attention [30]–[31]. These results do not contradict the present oscillation measurements. It should be also noted that spatial distribution was clearly different between beta oscillation and network measure (global hub) in this frequency.

## Conclusions

The present study revealed task-related changes in functional properties of the human brain with reduced functional segregation, while global hubs were found in prefrontal and sensorimotor areas. The present study is thus the most fundamental first step to examine task-related rapid changes in functional network structures associated with a variety of brain functions.
